# Raising employment and quality of life among people with disadvantages – results of a Hungarian project

**DOI:** 10.1186/s12889-021-11763-z

**Published:** 2021-09-23

**Authors:** Noémi Meisznerné Kuklek, Máté Cséplő, Eszter Pozsonyi, Henriette Pusztafalvi

**Affiliations:** 1 Faculty of Health Sciences, Doctoral School of Health Sciences, University of Pécs, Pécs, Hungary; 2 Department of Health Promotion and Public Health, Faculty of Health Sciences, Institute of Health Insurance, University of Pécs, Pécs, Hungary

**Keywords:** Disadvantage, Disability, Labor market program, Mental health, Mental well-being

## Abstract

**Background:**

People with disadvantages are a high-risk group of unemployment or underemployment. Disadvantages include disability, under-education, or being a member of a minority, etc. Effective labor market programs could be a key in raising employment and quality of life among this high-risk group of society. The TOP 6.8.2.-15-NA1 project is one of the main Hungarian labor market programs. The project’s primary aims are increasing the employability of disadvantaged unemployed and supporting the efficiency of job-seeking.

**Methods:**

Our goal was to analyze the effects and methodology of the TOP 6.8.2.-15-NA1 project in Hungary. The sample of our study contains participants of the project (*n* = 300), based in Zala County, Hungary.

**Results:**

After 28 days, 53.3% of participants had a job. At the 180th day status, the rate of employed people was 47.3%. We could identify low-educated participants and older participants as higher-risk groups of long-term unemployment.

**Conclusions:**

We emphasize the role of these services (job-seeking clubs, organization of job fairs, and mentorship) in the long-term individual success of participants. Improving the employment rate for people with disadvantages is a critical factor for enhancing the quality of life for individuals with disadvantages.

## Background

After the economic depression in 2008, the social and economical processes have changed in the past few years. These changes pointed out that new strategies should be worked out to threaten the upcoming problems of the labor market. In the countries of the European Union, there is a general shortage of labor in some sectors, while others show oversupply [1].

Building up labor market systems, which can follow the structural changes of economies of European countries is an important goal of the European Union. Accordingly, it is essential to establish cooperation mechanisms.

The Europe 2020 strategy is the European Union’s agenda for economic growth and jobs for the actual decade. The strategy focuses on sustainable and inclusive growth to improve Europe’s competitiveness and productivity. It also emphasizes a sustainable social market economy. The goal of the Europe 2020 strategy was to reach a 75% employment rate among the 20–64 year-old population in Hungary [1].

According to the data of the European Commission, the employment rate was 70.1% in 2015 in the EU28 countries. Until 2019, this rate increased to 73.9, which is very close to the targeted 75% level. In Hungary, the employment rate was only 68.9% in 2015. This level significantly increased during the second half of the decade, reaching 75.3% until 2019. The increase in the employment rate was higher in the country, compared to the average increase in the EU28 [2].

This success of raising the official employment rate in Hungary has many different reasons. One of these reasons is the high efficiency of labor market programs, which were conducted in cooperation with the European Union.

Accordingly, studies focusing on these projects help to map the factors of effective programs, contributing to higher employment and better quality of life among people with disadvantages.

We aimed to map the indicators which can mark the effects of the project. We also aimed to describe the requirements of successful integration. The analysis focused on the results of the disabled subgroup.

According to several studies about unemployment and labour market programs in Central-Eastern Europe, both sex and age have a significant effect on long-term unemployment [3, 4]. For older workers is difficult to sustain employment. Usually, if unemployment appears at this age group, there is a one-way exit, in inactivity via invalidity pensions [4, 5]. Long-term unemployment appears to have a stronger impact on men’s lives than women, men are much more stigmatized than women for their unemployed status and spend more time looking for a job. Accordingly, for men, it is easier to find a job than for women in the region [4]. In the case of both sexes, improving skills and labour market services proved to be helpful in finding a long-term job [3–5].

Based on these experiences [3–5], the study aimed to test the following hypotheses:

1. Sex and age correlate with the chance of individual success.
2. A significant difference in individual success can be established between the target groups.
3. The length of the service does not correlate with individual success.

### People with disadvantages in the labor market

Disability is one of the main disadvantages in the labor market. It has several different types, such as those that affect an individual’s vision, movement, communication, quality of life, social relationships, etc. The subgroup of people with disabilities is a diverse group with a wide range of different needs and problems. According to the World Health Organization (WHO), disability has three main dimensions [6]:

1. Impairment in a person’s body structure or function, or mental functioning (loss of vision or memory, loss of a limb)
2. Activity limitation (difficulty seeing, hearing, problem-solving, etc.)
3. Participation restrictions in normal daily activities (working, engaging in social and recreational activities, etc.)

There is a strong correlation between the quality of life and disability among adults [7, 8]. Psychological distress is also high among this subgroup [9]. Among men, disability is mainly discordant with their masculine identity and its main component, self-reliance. This component is more important to men with disabilities. It is also associated with mental well-being problems among adult males. Disadvantages also include undereducation [8, 9].

People with disadvantages, and especially in Hungary, members of Romani people are high-risk groups of both health problems and unemployment or underemployment [10]. This association exists in the case of different types of disadvantages. According to a Swedish study [11], people with mobility disabilities are vulnerable groups at risk of prolonged unemployment during their lifetime, also in a country with a good welfare system.

This association between disadvantage and lack of work is also true in younger populations. Using a causal approach, Harkko et al. [12] suggest that unemployment is consistently associated with an increased risk of disadvantage. The reason behind this correlation is the higher risk of common mental disorders among young unemployed people.

According to a review about unemployment’s effects on health [13], long-term unemployed people carry a higher risk of mental illness, compared to employed people. The risk of mental health problems increases with the duration of unemployment status. Although employment carries risk of stress and frustration [14, 15], unemployment status also correlates with lower quality of life through lower socioeconomic status, mental well-being, disadvantage, access to education and health care, etc. [12, 14, 15]

In addition, people with disadvantages are more probable to experience underemployment and a higher risk to have their mental health negatively affected by this status [16].

Mediator variables between mental health and unemployment/underemployment include stress, depression, and unhealthy behaviors. Unemployment also correlated with a higher risk of later life with disadvantages. On the other hand, only a few studies focused on the correlation between total lifetime unemployment with disadvantages and life expectancy [17, 18].

According to the HILDA (Household, Income and Labour Dynamics in Australia) survey, which included 2379 participants with disadvantages, a greater reduction in mental health was established for the unemployed or inactive people with disadvantages compared to people who were employed. The study highlighted the value of employment for people with disadvantages [16].

This negative effect of unemployment on mental health can be broken only by mental health-promoting programs among the unemployed with disadvantages, and special labor market projects focusing on high unemployment risk groups [16, 18]. The latest data on this field shows some positive examples. During the Healthy People 2020 project in the US, notable progress could be reached in case of access to services for complex conditions associated with disadvantage, expansion of mental health promotion programs focusing on disability, and reducing the unemployment rate among job seekers with disadvantages [19].

In summary, unemployment among people with disadvantages is an important determinant of their poor mental health. Governmental policies should oddly focus on this extremely high-risk group, to help their integration into the labor market. Studying and interpreting the positive examples of labor market integration programs are oddly important to support this process.

### The TOP project

The TOP 6.8.2.-15-NA1 (local employment cooperation in urban areas, TOP project) is one of the main Hungarian labor market programs. Its main aims are to provide manpower according to the needs of the labor market, to increase the employability of disadvantaged, unemployed, and inactive people who are willing to work in the region, to support the efficiency of job-seeking, and to lead public employees to the labor market [20, 21].

The goal of the TOP project was to build up a local strategy based on the analysis of the labor market and innovation options. Educational and employment programs were conducted based on this strategy, for the purpose of the labor market integration of unemployed and disadvantaged people. During the period of the project, the local employment pact and the pact office were also established. The project was conducted with the collaboration of the Employment Office of Zala County and the Nagykanizsa Employment Non-profit GmbH. The main goal was to help at least 300 unemployed people in Nagykanizsa, Hungary finding jobs by using the established labor market services.

The TOP project also includes innovations and new methods, which can be used in labor market policy both locally and nationally. It has 10 different target groups (Table 1).

**Table 1:** Target groups of the project

Target groups: unemployed people, employees of the public sector, and inactive people	
Disadvantaged people according to the project:	
1.	People with a low level of education (elementary school or below)
2.	24-year-old or younger people or 29-year-old or younger unemployed people
3.	50+ year-old people
4.	Women returning to the labor market after giving birth
5.	People receiving unemployment benefits
6.	People at risk of permanent unemployment
7.	People with disabilities
8.	Members of the Romani minority
9.	People who are capable to be led from public employment to the labor market
10.	Inactive people

The TOP project includes an individual program. The elements of this program contain job advice, job-seeking clubs, organization of job fairs, and mentorship. Providing services had two different ways. The first option was that the service as an individual element of the project. The second type was when the service was conducted in connection with the participant’s support. During the services, participants had to document the completion. In practice, the document of completion included a written reference by the participant, the mentorship diary, the diary of progression, and the mentor’s evaluation of the participant’s activity [20].

## Methods

### Selection and description of participants

The sample of our study contained people, who were participating in the TOP 6.8.2.-15-NA1 project (*n* = 300). We have studied the efficiency of employment policy tools from the aspect of labor market integration. The study included 1.5 years.

### Statistics

#### Input measures

The study used input and output variables to measure the efficiency of the TOP project.

We have used the sociodemographic measures of *age and sex* as input variables.

The target group of the participant was also used, which contained the 10 *target group*s of TOP project as possible responses. Each job seeker is assigned to one of the categories (Table 1) when they start participating in the TOP project. The recommended services are assigned to each of the categories in the Individual Action Plan (IAP). In addition, caseworkers can offer all services to any jobseeker, even if these services do not fall under the recommended set of measures [20].

The length of the service (in days) was also a numeric input variable in the study. In addition, we have also included the length of external service.

#### Output measures

The study uses several variables as output measures, which were based on the individual success of the participants.

The first measure is the ratio of participants who *finished the project,* which is also an output variable of the study. The two categories of the dichotomous variable were ‘finished successfully’ and ‘withdrawn unsuccessfully’.

After finishing the project, the status of the participants was also monitored. They had to fill a form on the 28th and 180th day after completing the project. The *employment status after 28 and 180 days* had the following possible responses: ‘employed or self-employed with support’; ‘employed without support’; ‘self-employed without support’; ‘public employment’ and ‘unemployed’. Employed status in 28 and 180 days also included public employment, but only with an acceptable quality of the individual program and mentored placement. The variables were coded into dichotomous variables with two categories, like ‘employed’ and ‘unemployed’ (which included public employment).

### Statistical analysis

Chi-square tests and binary logistic regression were used to test the associations between the included variables. IBM SPSS (Statistical Package for the Social Sciences) 23 software was used to conduct the analyses.

## Results

According to descriptive statistics (Table 2), the mean age of the sample was 45.51 (21–66). Females were slightly overrepresented among the participants (58.3%). The average length of service was 197 days among the participants of the program. Almost one-third of participants were categorized as members of target group No. 3. (50 years old or above), while 53 people were at risk of permanent unemployment (No. 6.). The third-largest target group (No. 1., low-educated group) contained 36 participants. In total, a very high rate of participants (99.3%) finished the TOP project successfully. After 28 days, 160 of them had a job (53.3%). At the 180th day status, only 246 participants filled the form about their job status. Among them, the number of employed people was 142 (47.3% of all participants; 57.7% of participants answered). We could observe a nominal decrease, but a rise in employment rate between the 28th and 180th day status.

**Table 2:** Descriptive statistics of input and output measures (*n* = 300)

Input variables	n (%)	Mean (SD)	Output variables	n (%)
Age		45.51 (11.34)	Finished successfully	298 (99.3%)
Sex			Employment status after 28 days	
Male	125 (41.7%)		Employed	160 (53.3%)
Female	175 (58.3%)		Unemployed	134 (44.7%)
Target group			Employment status after 180 days	
No. 1.	36 (12%)		Employed	142 (47.3%)
No. 2.	17 (5.7%)		Unemployed	104 (34.7%)
No. 3.	93 (31%)			
No. 4.	15 (5%)			
No. 5.	34 (11.3%)			
No. 6	53 (17.7%)			
No. 7	2 (0.7%)			
No. 8.	18 (6%)			
No. 9.	7 (2.3%)			
No.10.	25 (8.3%)			
Length of service (days)		197 (132.42)		

According to the results of binary logistic regression (Table 3), age correlated with both 28th day (OR = 0.97; CI95% = 0.95–0.99; *p* < 0.05) and 180th day (OR = 0.96; CI95% = 0.94–0.98; *p* < 0.01) employment status negatively. Accordingly, older participants had less chance to find both a short-, and long-term job after the project.

**Table 3:** Binary logistic regression of input variables and individual success, *n* = 300

Predictor variables	Employed after 28 days		Employed after 180 days	
	OR	CI95%	OR	CI95%
Age^a^	0.97*	0.95–0.99	0.96**	0.94–0.98
Sex				
Female^b^	1		1	
Male	1.17	0.72–1.90	1.45	0.85–2.49
Length of service (days)^a^	1.01***	1.01–1.01	1,01**	1,01 – 1,01

^a^Continous variable

^b^Reference category

*p < 0.05

**p < 0.01

***p < 0.001

In case of sex, no correlation was established with the two included output variables. Length of service had a positive correlation with employment status, the association was significant in case of both 28th day (OR = 1.01; CI95% = 1.01–1.01; *p* < 0.001) and 180th day (OR = 1.01; CI95% = 1.01–1.01; *p* < 0.01) status. This result highlights the positive effect of the project’s services.

According to its almost homogenous distribution, the variable of successfully finishing of the project was excluded from the analysis.

A significant difference was found among the target groups of TOP project and individual success in finding a job in 180 days (Table 4). On the other hand, no correlation was established with employment status after 28 days. The worst rates of successful job seeking were observed in groups No. 1. (low educated group), No. 3. (50 years old or above), and No. 6. (people at risk of permanent unemployment).

**Table 4:** Chance of finding a job after the project among the target groups, *n* = 300

Target group	Employed after 28 days (*n* = 294)	Employed after 180 days (*n* = 246)*
No. 1.	38.2%	38.7%
No. 2.	43.8%	41.2%
No. 3.	37.4%	32.1%
No. 4.	60%	61.5%
No. 5.	44.1%	50%
No. 6	43.4%	34.9%
No. 7	50%	50%
No. 8.	70.6%	100%
No. 9.	28.6%	50%
No.10.	72%	64.3%

* p < 0.05; chi-square test

## Discussion

The TOP project had a very high finishing rate in all target groups, only 2 out of the 300 participants failed to finish the program. The project had an overall positive effect on the employment status of the participants. The prompt effect (after 28 days) was more positive compared to the long-time effect (180 days), but it is also acceptable (nearly half of participants could find and keep up a long-term job).

A negative association between age and individual success was established. Accordingly, we could verify our first hypothesis. Older age groups should be considered as higher risk groups during labor market programs.

We could also verify the second study hypothesis, which presumed a difference between target groups and success in job seeking. Short-term (28 days) success did not differ among the target groups, but long-term success did. We could identify low-educated participants, older participants (50 years old or above), and people at risk of permanent unemployment as higher-risk groups of long-term unemployment after the project. Other studies also established that these groups are at a high risk of unemployment after participating in a labor market project [22, 23].

The third hypothesis could also be verified. The length of the provided services and education had a positive correlation with individual success in finding a long-term job after the program. Latest findings on services of labor market programs also establish the same result [23]. Accordingly, labor market projects should focus on the length and quality of the provided education and services.

## Conclusions

There are substantial costs to both individuals and society related to poor employment outcomes for people with disadvantages, including working disabilities. Improving the employment rate for people with disadvantages is a critical factor for enhancing the quality of life for individuals with disadvantages, and their families.

Among TOP project participants, we could identify low-educated, older participants and people at risk of permanent unemployment as higher-risk groups of long-term unemployment after the project. The fact that only a minority of these groups could find a long-term job is a limitation of the project. We emphasize the main role of the project’s services (job-seeking clubs, organization of job fairs, and mentorship) in the long-term individual success of participants. In the future, labor market programs should add special activities for older participants, such as training classes on computer and internet use.

Limitations of the study should also be mentioned. Only a few independent variables were used in the model, which is too simple to rule out confounders. This means that findings are possibly not with reliability.

In summary, several participants have been employed and were provided with education during the program. Thanks to the project, new jobs also became available. The increasing level of employment rate in these disadvantaged subgroups has several benefits for the country’s economy and the individual’s quality of life. Firstly, a direct positive impact on GDP could be observed after the rising rate of employment among individuals with disadvantages. In addition, an indirect impact could also be established via improved government fiscal balances. Positive effects also include the broader welfare gains for the individuals that secure employment, and their families and carers. In the local area of the study, a decrease in inactivity, increase in wages, and rate of female employment could be the indirect effects of the project.

Based on the findings, policymakers need to focus on increasing the job-seeking skills for people living with a disadvantage. Labour market programs proved to be effective in fighting against long-term unemployment. Thus, policymakers should give more attention to these programs. Age had a highly significant, negative effect on reemployment probability. Older people, especially with a disadvantage, have usually no option but inactivity via invalidity pensions. Reintegration of this high-risk group into the labour market would have a very positive effect both economically, socially, and individually [4, 22, 23]. Accordingly, government policy should give special attention to older unemployed people. The current instruments of supporting young people in the labour market (tax deduction, travel contribution, etc.) [4, 5] should be expanded, involving older unemployed people, especially with disadvantages.

## Data Availability

The datasets used and/or analyzed during the current study are available from the corresponding author on reasonable request.

## Abbreviations

HILDA: Household, Income and Labour Dynamics in Australia
IAP: Individual Action Plan
SPSS: Statistical Package for the Social Sciences
TOP project: TOP 6.8.2.-15-NA1 project
WHO: World Health Organization
